# Comparison of the Hematological Profile of Elite Road Cyclists during the 2010 and 2012 GiroBio Ten-Day Stage Races and Relationships with Final Ranking

**DOI:** 10.1371/journal.pone.0063092

**Published:** 2013-04-30

**Authors:** Giovanni Lombardi, Patrizia Lanteri, Pier Luigi Fiorella, Luigi Simonetto, Franco M. Impellizzeri, Marco Bonifazi, Giuseppe Banfi, Massimo Locatelli

**Affiliations:** 1 Laboratory of Experimental Biochemistry and Molecular Biology, I.R.C.C.S. Istituto Ortopedico Galeazzi, Milano, Italia; 2 Commissione Tutela della Salute - Federazione Ciclistica Italiana, Roma, Italia; 3 Department of Medicine, Surgery and Neuroscience, University of Siena, Siena, Italia; 4 Department of Biomedical Sciences for Health, University of Milano, Milano, Italia; 5 Diagnostica e Ricerca San Raffaele, Ospedale San Raffaele, Milano, Italia; Wayne State University School of Medicine, United States of America

## Abstract

Cycling stage races are strenuous endurance events during which exercise-induced variations in hematological parameters are consistently observed. However, specific literature on such changes is scarce and published data have been derived from small samples of athletes. The aims of this study were: (1) to determine the hematological response to middle-term strenuous endurance; and (2) to determine whether a relationship exists between the athlete-specific hematological profile and final placement in a cycling stage race. The study population was male professional cyclists (n = 253) competing in the 2010 (n = 144) and 2012 (n = 109) GiroBio 10-day stage races. Blood draws taken before the start of the race, at mid-race, and at end-race were performed in strict compliance with academic and anti-doping pre-analytical warnings. Blood chemistry included white blood cell, red blood cell, hemoglobin concentration, hematocrit, mean corpuscular volume (MCV), mean hemoglobin content (MCH), mean corpuscular hemoglobin content (MCHC), platelets, and reticulocyte relative and absolute counts. Compared to baseline values, erythrocyte, hemoglobin, hematocrit, MCHC, platelet and reticulocyte counts were all consistently lower at mid-race, but returned to normal by race-end, while leukocytes were increased in the final phase. MCV increased during both events. MCH increased in the first part to then return to baseline in the 2012 race. The calculated OFF-score consistently decreased in the first half of the race before increasing, but remained lower than the baseline value. The trends of variation in hematological parameters were substantially similar in both events. There was an inverse, albeit weak, relationship between placement and erythrocyte, platelet, hemoglobin, hematocrit and OFF-score values in the 2010, but not in the 2012 race. In conclusion, the data confirm that, in this large series of elite road cyclists, the strenuous effort a rider sustains during a stage race induces appreciable changes in the hematological profile.

## Introduction

Cycling stage races are among the most strenuous of endurance events. The exercise-induced variations observed in hematological parameters appear to be consistent with the rider’s physiological response to maintain and improve highly demanding performances day-after-day [1], [2]. During training and competition, an essential part of evaluating the health and performance of professional and recreational athletes is periodic assessment of the hematological profile. Together with evaluation of iron metabolism, serial blood chemistry analysis can point to whether an out-of-range shift in blood parameters may be attributable to the response to physical effort or to an index of abnormal response [3], [4], [5].

Despite media exposure of doping scandals and subsequent disciplinary action taken by sports organizations against cheating athletes, efforts to restore cycling’s tarnished reputation have been thwarted by the illicit conduct of individual athletes; sometimes acting with their physician’s complicity. Being an endurance sport, cycling is pervaded by the use of ever more sophisticated doping procedures that increase oxygen delivery to muscles. Erythropoiesis-stimulating agents and autotransfusion have been the most frequently detected performance-enhancing substance and procedures [6].

A starting point for determining irregular and suspect behavior in athletes is a better appreciation of the hematological response to vigorous physical activity. This is of particular interest in the context of the Athlete’s Biological Passport (ABP), which was devised to detect abnormal variation(s), even at a single time-point, versus a subject-specific physiological range deduced from the athlete’s own previous data [7], [8]. Because the variations during a competitive season affect the behavior of hematological parameters over a season, knowing their variability could help to define the physiological ranges in an athlete [4].

The GiroBio, held in mid-June every year in northern Italy, is the “under-27 s amateur Giro d’Italia”, a surrogate for the Giro d’Italia and other international road races (Tour de France, Vuelta a Espana) for young cyclists. It attracts more than 150 professional cyclists from all over the world annually. Since 2005 it has been included in the Union Cycliste Internationale (UCI) Europe Tour circuit, category 2.2. About half the duration of its major counterpart, the GiroBio format is 10 stages over 11 days. The GiroBio race represents the entry step to a fully professional career for most cyclists. The race aims to promote the values of sport and fair play in healthy competition, counteracting the doping culture, through the adoption of innovative organizational aspects, as discussed below.

To our knowledge, few studies have analyzed the hematological profile of cyclists competing in a cycling stage race. All prior studies had small populations, mainly from one or two teams, because of the difficulties inherent in organizing such an evaluation by a non-official, anti-doping agency. Recent findings reported by our own group demonstrated that major metabolic modifications in homeostasis, systemic and organ and tissue-specific, occurred in response to the metabolic effort cyclists sustained during the 3-week Giro d’Italia stage race [9].

In this study, we evaluated the hematological profiles of 253 elite cyclists competing in the 2010 and 2012 GiroBio races. The aims were; (1) to define a general trend in the variations of the hematological parameters, including those relevant for anti-doping purposes, in response to middle-term strenuous endurance; and (2) to determine whether a relationship between the athlete-specific profile and final placement in both race events is present.

## Materials and Methods

### Ethics Statement

The study was approved by the National Commission on Health Protection [Commissione Nazionale Tutela della Salute], the Italian Cycling Federation [Federazione Ciclistica Italiana, Rome, Italy] and the Department for Youth Policy and Sport – Presidency of the Council of Ministers [Dipartimento per le politiche giovanili e le attività sportive - Presidenza del Consiglio dei Ministri, Rome, Italy]. The team participation was on a voluntary basis upon a request to the organizers. Nevertheless, race participation required the cyclists and team staffs to follow the competition’s organizational model. Sanction for any infractions of the competition rules was the disqualification from the race of the cyclists and/or team. A written informed consent for using the data collected during the race for research purposes was signed by all subjects. The clinical investigations were conducted according to the principles expressed in the Declaration of Helsinki.

### Study Population

Of the 346 male professional cyclists competing in the 2010 and 2012 GiroBio events and recruited for this study, 253 riders completed the race and had blood drawn. The present analysis included only the data for the subjects from whom all three blood samples were collected: 144 out of 178 riders competing in the 2010 GiroBio, and 109 out of 168 riders competing in the 2012 GiroBio. The mean age of the riders in the 2010 GiroBio was 21.8±1.7 years and 22.3±1.6 years in the 2012 GiroBio. Nineteen riders competed in both the 2010 and 2012 races.

Both GiroBio events were held, as usual, in mid-June; the total length covered was 1,276.9 km in 2010 and 1,351.5 km in 2012. The difference in elevation for each stage was calculated from the point-by-point sum of the difference in altitude. The main features of each stage of the two races are illustrated in Table 1.

**Table 1:** Stage features, climatic conditions, and study design of the 2010 and 2012 GiroBio events.

	GiroBio 2010						GiroBio 2012						
Day	Stage	Kind of Stage	Length (km)	Difference in Height (m)	BloodDrawn	External Temperature °C (start-finish)	Stage	Kind of Stage	Length (km)	Difference in Height (m)	Blood Drawn	External Temperature (°C) (start-finish)	
**−2**	/	/	/	/	T1	22.0	/	/	/	/	T1	22.0	
**−1**	/	/	/	/	/	19.4	/	/	/	/	/	22.0	
**1**	1	LM	111.6	2820	/	19.4–21.0	1	F	144.0	1556	/	23.0–31.0	
**2**	2	F	168.9	3615	/	23.0–29.0	2a	F	75.6	642	/	25.0–30.0	
							2b	TT	12.1	0	/		
**3**	3	F	155.5	198	/	25.0–29.0	3	LM	193.3	2886	/	23.0–28.0	
**4**	4	HM	154.2	3028	/	24.0–29.0	4	LM	168.4	2703	/	24.0–9.0	
**5**	5	LM	184.1	2818	/	21.0–21.0	5	LM	156.0	2136	/	22.0–23.0	
**6**					T2	23.0	Rest	/	/	/	T2	20.0	
**7**	6	F	148.2	69	/	19.0–26.00	6	LM	163.8	2331	/	20.0–24.0	
**8**	7	TT	30.5	493	/	22.0–26.0	7	F	147.1	1126	/	24.0–24.0	
**9**	8	LM	170.9	3300	/	21.0–22.0	8	HM	168.2	5190	T3	25.0–21.0	
**10**	9	LM	153.0	2367	T3	17.0–21.0	9	HM	123.0	4990	/	27.0–34.0	
**Tot**			1276.9	18168					1351.5	23560			

The table summarizes the features of the two races. F: Flat, TT: Time-trial, LM: Low Mountain, HM: High Mountain; T1: Pre-race blood drawn, T2: mid-race blood drawn, T3: end-race blood drawn. The reported environmental temperatures refer to 8∶00 a.m. (time of blood sampling) for the pre-race and rest days and to the start and finishing time for each day of a stage.

On the basis of an innovative organizational and managerial model for the race, all riders stayed in the same accommodations, they received the same diet (about 5,700 kcal per day per rider, composed of 15% protein, 24% lipids and 61% carbohydrates) and standardized medical care and dietary supplementation between meals were also provided, in compliance with health and safety protection rules as established by Italian law. Finally, all the riders were followed by the same medical-scientific team.

### Assessment of Hematological Profile

Under the provisions of the race regulations, participants’ health status was assessed by blood testing prior to the start of the race, halfway through the race and at the end; that is, at day -2 (T1), day 6 (T2), and day 10 in the 2010 event, and day 9 in the 2012 event (T3). The academic-accepted pre-analytical warnings [10] and official rules of the World Anti-Doping Agency (WADA) [11] and Union Cycliste Internationale (UCI) [12] concerning blood sample collection and sample transport were strictly followed. Briefly, standard blood draws were taken in the morning between 08^00^ and 10^00 ^h, after overnight fasting; after awakening, the riders rested in a sitting position for at least 10 minutes with feet on the floor before blood was drawn [11]. Blood was collected into 3.5 mL evacuated tubes coated with K_2_EDTA (BD Vacutainer Systems, Becton–Dickinson, Franklin Lakes, NJ, USA). Immediately after drawing, the tubes were inverted ten times and stored at 4°C in a temperature-controlled portable electric refrigerator, and then transported by car to the laboratory for analysis. Despite the distance between sampling site and laboratory, the time between sampling and analysis was always less than 12 hours. On arrival at the laboratory, the K_2_EDTA-anticoagulated blood was homogenized for 15 min prior to analysis, in compliance with UCI and WADA recommendations [11], [12].

Blood chemistry included: white blood cell count (WBC 10^9^/L); red blood cell count (RBC 10^12^/L); hemoglobin concentration ([Hb], g/L); hematocrit (Ht, %); mean corpuscular volume (MCV, fL); mean hemoglobin content (MCH, pg); mean corpuscular hemoglobin content (MCHC, g/L); platelet count (Plt, 10^9^/L); reticulocyte relative count (Ret%, %); and reticulocyte absolute count (Ret#, 10^12^/L). Analyses were performed on a Sysmex XE 2100 (Sysmex, Kobe, Japan); the imprecision of the hematological tests was <1.5%, except for Ret% which has an imprecision <15.0%. Internal quality controls were performed by the means of e-Check (LE Tri level, Sysmex, Kobe Japan). Instrumentation was also controlled during the study period by external proficiency testing.

### Statistical Analysis

The OFF-hr score (OFF-score) was calculated as follows [13]:

OFF-score = [Hb] - 60√Ret%.

with [Hb] expressed as g/L. This score is used to detect blood doping; for example, an OFF-score higher than 110 indicate a probability of 99.9% that blood manipulations have been carried out, even many weeks before.

Statistical analysis was performed using SPSS version 14.0 (SPSS Inc., Chicago, IL, USA). Normal distribution of values was tested using the Kolmogorov-Smirnov normality test; in the descriptive analysis, all normally distributed values are expressed as the means ± SD while data from non-normal distributions are expressed as the median and the distribution range. A repeated measures one-way analysis of variance (ANOVA), with Bonferroni’s correction for post-hoc analysis, was used to compare normal data over time; Friedman’s test with Dunn’s correction for post-hoc analysis was used to compare data from non-normal distributions over time. Wilcoxon’s matched pairs test was used to compare the values for each time-point in the two races.

Correlations among hematological parameters and between hematological parameters and ranking were examined using Spearman’s rank correlation coefficient. Furthermore, to detect differences in the hematological profile in relation to the final ranking, the profiles of the cyclists completing the race in the first 30 positions were compared with those of the last 30 positions: t-tests were used to compare each time-point, for each parameter, between the two groups and to compare the differences, “Δ”, between T2 and T1, T3 and T2 and T3 and T1, between the two groups. The significance level was set at 0.05.

## Results

### Variations between Races

**Figure 1:**
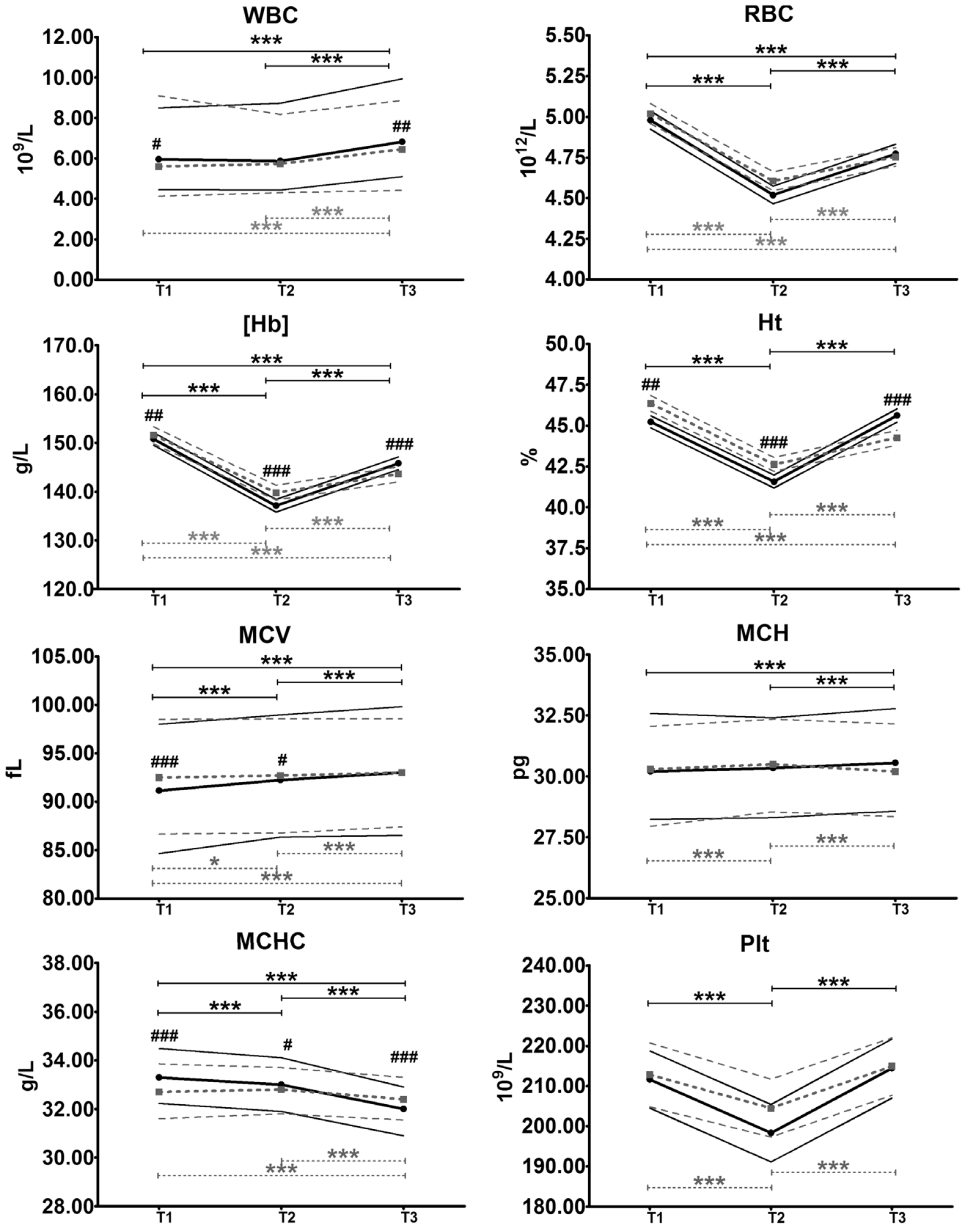
Hematological profile trends during the two races. The figure shows the trends of white blood cells (WBC), red blood cells (RBC), hemoglobin concentration ([Hb]), hematocrit (Ht), mean corpuscular volume (MCV), mean hemoglobin content (MCH), mean corpuscular hemoglobin concentration (MCHC), platelets (Plt) during the 2010 (black • and solid line, n = 144) and the 2012 (grey ▪ and dotted line; n = 109) races. The thinner lines (solid for 2010 and dotted for 2012) represent the 5^th^–95^th^ percentile for WBC, MCV, MCH and MCHC, while for RBC, [Hb], Ht and Plt they represent the 95% confidence intervals. The asterisks (*) indicate significant differences between different time-points in the same race (*: p<0.05; **: p<0.01; ***: p<0.001). # indicates that the value of the time-point in the 2012 race is significantly different from that recorded in the 2010 event (##: p<0.01, ###: p<0.001).

Comparison of the data from the two races showed consistent changes in the riders’ hematological profile. WBC increased only in the second half, while RBC, Hb, Ht and Plt decreased in the first half before increasing thereafter. Hb decreased by about 8.5% at T2, 4.3% at T3 versus T1. MCV increased and MCHC decreased; only MCH constantly increased during the 2010 race, while it first increased and then decreased during the 2012 event. The results are summarized in Figure 1.

**Figure 2:**
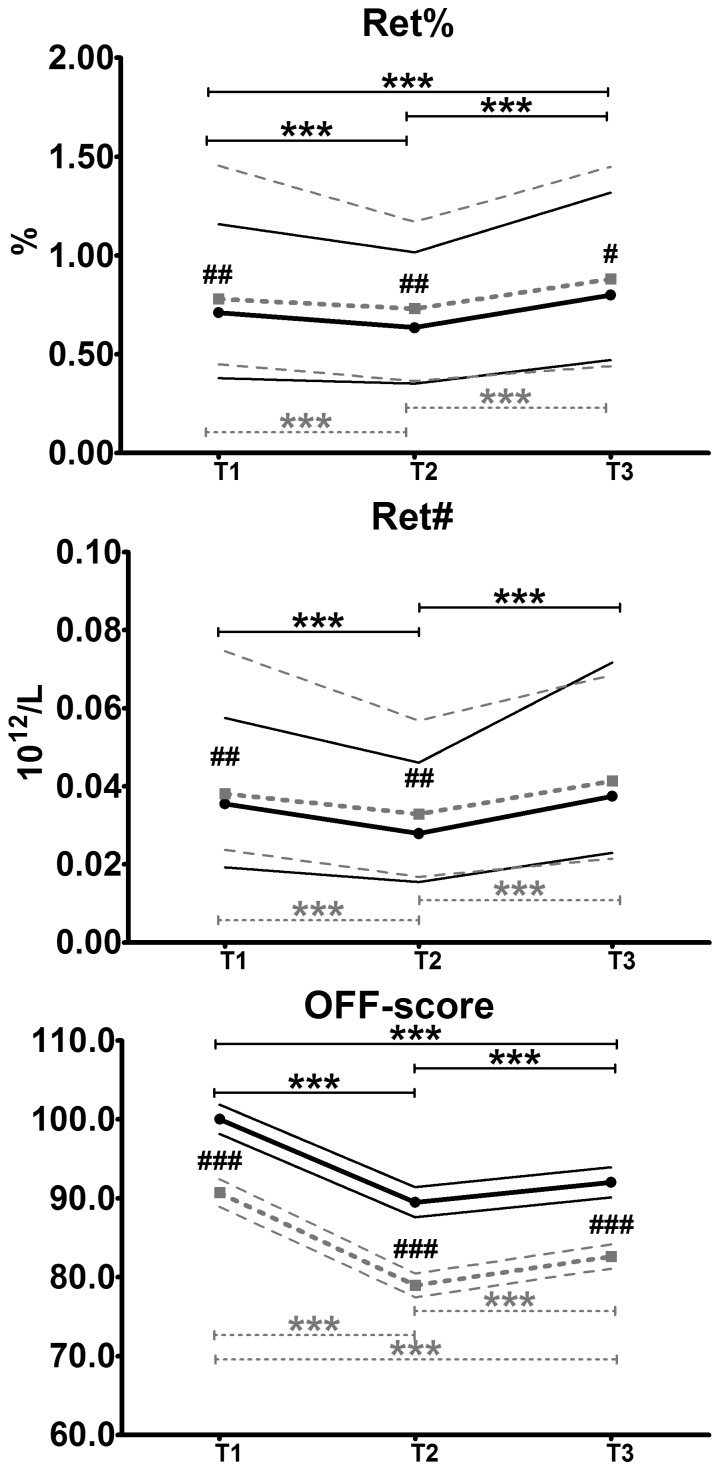
Reticulocyte counts and OFF-score trends during the two race events. The figure shows the trends of Ret relative count (Ret%), Ret absolute number (Ret#) and OFF-score during the 2010 (black • and solid line, n = 144) and the 2012 (grey ▪ and dotted line; n = 109) races. The thinner lines (solid for 2010 and dotted for 2012) represent the 5^th^–95^th^ percentile for Ret% and Ret#, while for the OFF-score they represent the 95% confidence intervals. The asterisks (*) indicate significant differences between different time-points in the same race (*: p<0.05; **: p<0.01; ***: p<0.001). # indicate that the value of the time-point in the 2012 race is significantly different from that recorded in the 2010 event (##: p<0.01, ###: p<0.001).

The data from both races showed a U-shaped trend in reticulocyte count. Ret% decreased by about 6% from T1 to T2 and then increased by about 14.5% at T3, as averaged between the two races. Accordingly, the Ret absolute number significantly decreased by about 18.5% in the first half of the race and then increased by about 29.5% thereafter. In general, the calculated OFF score followed the same trend as the other hematological parameters. The results of Ret count and OFF-score are reported in Figure 2.

In order to define the physiological significance of the time-dependent variations, the theoretical intra-individual variability (CV_Bw_) [14], [15] and the percentage change were compared for each parameter, as obtained by the paired comparison of the time-points (T2 vs. T1, T3 vs. T2 and T3 vs. T1). As shown in Table 2, comparison of subject-specific modifications reveals a very high number of riders with intra-individual variations exceeding the theoretical values.

**Table 2:** Comparison between the theoretical biological variation and the intra-individual variations recorded in the study cohorts.

		2010 (subjects exceding CV_Bw_)			2012 (subjects exceding CV_Bw_)		
	CV_Bw_ (%)	T2vsT1	T3vsT2	T3vsT1	T2vsT1	T3vsT2	T3vsT1
		(n = 144)	(n = 144)	(n = 144)	(n = 109)	(n = 109)	(n = 109)
**WBC**	11.2	61	95	92	55	58	71
**RBC**	2.1	141	126	112	104	83	92
**Hb**	3.4	136	118	81	95	56	77
**Ht**	2.4	134	137	87	106	81	81
**MCV**	1.3	60	44	104	28	16	36
**MCH**	1.6	21	40	38	26	19	18
**MCHC**	1.7	46	128	140	25	28	38
**PLT**	9.0	67	63	59	70	72	76
**Ret%**	13.0	93	106	92	75	80	67
**Ret#**	13.0	98	117	93	80	89	72

The table shows the theoretical intra-individual variability values (CV_Bw_) and the number of subjects (out of 144 for the 2010 race and 109 for the 2012 race) for each GiroBio race event, whose percentage variation between time-points exceeded the theoretical CV_Bw_.

### Comparison between the Two Races

Paired comparison of the same time-points in the two races revealed important differences. Taking the 2010 event as reference, the data from the 2012 race showed lower WBC counts at T1 and T3, but not in RBC, Plt or MCH. Hb and MCHC were both higher at T2 and lower at T3; Ht was higher at T1 and T2 but lower at T3. MCV was always higher (Figure 1).

The Ret counts were higher in 2012 than in 2010, except for Ret# at T3, which did not differ. Because of the differences in Hb and Ret%, the OFF-scores were significantly higher at all time-points in 2012 (Figure 2).

The differences in these comparisons aside, the trend for each parameter was generally similar. However, some exceptions were found when statisitical significance was considered: MCH remained constant in the first half of the 2010 race and increased thereafter, while it rose in the first half and then decreased in the 2012 event.

### Correlation between Hematological Parameter Values and Ranking

Spearman’s rank correlation analysis highlighted significant correlations between the riders’ final finishing positions and the value of some of the parameters in the 2010 event alone. At the end of the 2010 race, the RBC count, Hb concentration and Ht were inversely, albeit weakly, related with finishing position (r = −0.27, r = −0.27, r = −0.29, respectively; p<0.01), as were the Plt count (r = −0.27; p<0.01) and the OFF score (r = −0.29; p<0.001). That is, the higher the finishing position, the higher the values of the analyzed parameters. These relationships were completely lost in the 2012 race.

Comparison of the values recorded for the cyclists ranked in the first 30 and last 30 positions at each time-point revealed significantly higher values in the best compared to the worst performers for: RBC count, [Hb]**,** Ht, Plt count, and OFF-score (0.001<p<0.05). However, in the 2012 event, only the Ht values at T2 and at T3 were higher (p<0.05) in the best- versus the worst-ranked riders. Finally, no significant differences were found between the “Δ” (T2 vs. T1, T3 vs. T2 and T3 vs. T1) of the two groups in both races.

## Discussion

The widespread use of banned performance-enhancing substances and practices in elite-level cycling produces many headlines. With the stricter enforcement of anti-doping regulations (i.e., in- and out-of competition testing, legal action), however, the consensus is that attitudes have changed and that, though still present, doping is less common now than in the recent past [16]. The promotion of ethical values and the protection of athletes’ health must be the primary objectives in sports medicine [17].

Current knowledge of sports physiology and doping biomarkers is formalized in the Athlete Biological Passport (ABP) program: an algorithm tracking the longitudinal record of hematological parameters as a means to define an individual’s hematological profile and thereby identify potential deviations [17]. The central concept of the ABP is that a better appreciation of the physiological changes in the hematological profile related to training, competition, and altitude will allow discrimination of variations induced by illicit practices from those due to homeostatic response to physical activity [18]. The aim of our study was to add new information about the physiological response during a multiday cycling competition (stage race). To our knowledge, this is the first study to report the hematological variations in a large series of professional cyclists competing in two years of the same cycling stage-race.

In the last few years, the increasing number of published studies on variations in hematological parameters in elite athletes reflects the growing interest in the athlete’s physiological response to strenuous metabolic effort, especially in the context of the current debate on blood doping and the ABP [3], [19], [20]. However, few papers have focused on the physiological response to effort in cyclists and all of these studies involved small populations of elite athletes; mainly from one or two teams [9].

In a recently published study, in which 9 professional cyclists were followed over the 21-day 2011 Giro d’Italia stage-race, the authors highlighted that RBC, Hb and Ht decreased from baseline to the first half of the race before partially recovering, or “stabilizing”, by the end of the competition. No significant change in reticulocyte percentage was observed, although an upward trend was noted. No significant variation in any of the other parameters was recorded [9].

Previous studies have reported a decreasing trend in Hb and Ht over brief stage races [21] or during a part of long stage races (up to the 11th day) [22], [23], but these modifications were explained by the hemodilution that occurred during the race, which is a typical feature of endurance activity. Although erroneously reported in the original paper [9] and then subsequently corrected [24], after suggestions made by Gore et al. [25], Corsetti and colleagues did not find any significant, and univocal, changes in plasma volume. The authors attributed the modifications in Hb and Ht to the metabolic effort in which athletes were engaged [9].

During the 1984 Tour de France, 9 cyclists showed no variation in Hb and Ht measured thrice weekly [26], while Hb was stable in the first 10 days and decreased after 20 days in 15 cyclists competing in the Giro d’Italia [1]. In contrast, Hb was found to decrease at days 12 and 18, versus the pre-race values, in 9 cyclists during the 2007 Tour de France [2].

And while previously published studies reported analytical test results, none described the schemes for blood drawing, transport and storage, all of which can potentially affect the results [10]. Only in our previous work [9] was it stated that the blood samples were obtained and analyzed strictly following procedures and recommendations by official anti-doping agencies [11], [12]. Because the ideal conditions for correctly obtaining biological specimens were fulfilled in that study, it is used as a reference for the present discussion.

In the present study, we studied the modifications of blood parameters in a routine hematological profile in two large cohorts of elite cyclists, under 27 years of age, competing in the 2010 and 2012 GiroBio cycling stage races. To our knowledge, this cohort comprising a total of 253 subjects is the largest ever studied in cycling to date. Our main findings reside in the confirmation of previously reported trends [9]: RBC counts, Hb concentrations and Ht showed a consistent decrease during the first half of the race, versus resting values, and a subsequent recovery by the end of the competition. The same trend was observed for platelet count, while the WBC count was increased only in the final phase of the race, contrary to what was reported in the previous work [9]. Moreover, while Corsetti did not find any variation in the other parameters, we observed an increase in MCV and a convex trend for MCHC. Instead, MCH showed a constant increase in the 2010 event, but an initial increase followed by a return to baseline in the 2012 race.

Reticulocyte counts followed the same trend as that of RBC, Hb and Ht, in contrast to the previous study in which Ret% showed a rising, though not significant, trend. Schumacher and coworkers found that a short bout of 30–45 minutes of exhaustive exercise (treadmill or cycloergometer) induced, on average, a 0.5% increase in Ret% measured 10 minutes after the exercise in nearly all male and female endurance athletes. This trend was inverted after a prolonged period of intensive exercise, over the competitive season, with a decrease in Ret% of 0.1% [27].

In recent years, multivariate statistical models have been validated as reliable approaches to detect recombinant human erythropoietin (r-huEPO ) during current use (ON-model), as well as after recent use (OFF-model) [13]. Later research has focused only on the OFF-model, which is based on Hb concentrations and Ret%, because it has been claimed able to specifically detect recent blood doping practices, that are more likely to be discovered in unannounced anti-doping or intra-competition testing [28], [29]. Because of the variations in Hb and Ret%, the calculated OFF-score showed a consistent decrease from the resting value to T2 and a slight increase towards T3, however, still lower than the baseline value.

From a medical point of view, despite the statistical significance of some variations, not all of them necessarily play a physiologically relevant role. Indeed, it is unlikely that the significant variations observed in WBC (12–14%), MCV (0.2–1.0%), MCH (0.5–1.0%), MCHC (1.0–3.0%), and Plt (3.9–8.1%) have any physiological importance. On the other hand, the variation in RBC, Hb, Ht, and Ret count indicated noticeable adaptive modifications in the bone marrow microenvironment in response to the elevated metabolic demand. Similar Ret % behaviors were observed in cyclists during altitude training [3], [30], [31], [32].

Importantly, the changes in the most relevant parameters (both statistically and physiologically) often fell outside the range of biological variability, in terms of intra-individual variability [14], [15]. When we analyzed the percentage variations of the parameters for paired time-points, we found a very high number of subject-specific variations exceeding the established intra-individual variability. This finding confirms the hypothesis that vigorous metabolic effort effectively modified the hematological profile in these cyclists.

There were no evident discrepancies between the 2010 and the 2012 GiroBio events. While they tended to overlap, some differences did emerge between the two races when the riders’ final finishing position was correlated with the level of each parameter. In the 2010 race, inverse relationships, even though weak, were found between placement and RBC and Plt counts, Hb, Ht and OFF-score. This finding indicates that the athletes who achieved better positions had higher values, for those parameters. However, no such relationships were observed during the 2012 race. To confirm this possible relationship between finishing position and specific features of the hematological profile, we arbitrarily chose to compare the first 30 riders with the last 30. This was done to distinguish two cohorts (corresponding to about one fourth and one third of the total number of recruited subjects in the two race events, respectively) that could theoretically be considered different on the basis of clearly different results. When the values of each time-point between the first 30 riders and the last 30 were compared, the values of RBC, [Hb], Ht%, Plt and OFF-score were higher in the top group in the 2010 race, while only Ht% was found to be higher in the first versus the last riders in 2012. Taken together, these findings confirm the notion that, at least in endurance sports, a higher potential for oxygen transport (higher [Hb], RBC and Ht) is associated with improved performance [6]. Moreover, except for the climatic condition (similar in the two races), comparison of the characteristics of each event revealed some potentially pertinent differences: the total difference in altitude in the 2010 event was about 12,479 m in the first five stages (the first half of the race) versus 9,923 m in 2012. Furthermore, the third blood sample was drawn the day after the hardest stage in 2010, while in 2012 it was performed the day before the hardest stage. Hence, the differences in hematological behavior might also be attributed to the features of the race routes in addition to other, as yet undefined, factors.

Finally, the choice of the 2010 and 2012 events, but not the 2011 race, was dictated by the failure to hold blood collection in 2011. It should be noted that all the participants in the competitions were enrolled in the study, in keeping with the requirement of mandatory blood sampling carried out in the context of health status control. However, the analysis in this study was carried out using only the data derived from those subjects from whom all three blood samples were obtained. Therefore, the study population was not preselected.

The main limitation of the present study is that we cannot be absolutely sure that, despite all efforts to prevent it, blood doping may still have occurred. The pervasive use of illicit performance-enhancing substances and practices in cycling makes such suspicion warranted. In the GiroBio race, however, a major deterrent to the use of doping was the provision of common housing for all participants and the support of an independent medical team. Another possible limitation is that we studied the Hb concentration, whereas a recent study demonstrated that hemoglobin mass (Hb_mass_) measurements may be more useful. Indeed, the aim of most blood doping practices is to increase total Hb_mass_. Garvican and colleagues showed that, in contrast to Hb and Ht, the Hb_mass_ (measured via the CO-rebreathing test performed on location during a professional cycling tour) remained stable over 6 days. This finding indicates that Hb_mass_ has greater utility in ABP evaluation [33].

In conclusion, we found that, as compared to rest values, RBC, Ret and Plt counts, as well as Hb and Ht, all decreased after the first part of the race, before recovering at the end of the race in professional cyclists competing in two 10-day cycling stage races. The same trend was observed for the OFF-score. Finally, RBC, Hb, Ht and OFF-score values were inversely related to the riders’ final finishing position, though only in one of the two races. This finding confirms that the best performances are, at least in part, associated with higher potential for oxygen transport in blood. These data could be useful for defining a typical haematological profile that, in turn, can be used for identifying possible deviations caused by pathologies and abuse of illicit substances, or both. In fact, few papers have described, in a standardized way, the haematological behavior of athletes during such races. Moreover, international agencies, engaged in testing for blood doping have not released the values obtained from either negative- or positive-tested athletes. Thus, it is difficult to evaluate a regular profile based only on common haematological parameters. We acknowledge that further research in this area, together with the publication of haematological data from large cohorts of professional athletes, especially cyclists during competitions, is required to improve data interpretation in anti-doping evaluation.
